# Building a Foundation for Precision Onco-Nutrition: Docosahexaenoic Acid and Breast Cancer

**DOI:** 10.3390/cancers14010157

**Published:** 2021-12-29

**Authors:** Henry J. Thompson, Elizabeth S. Neil, John N. McGinley, Vanessa K. Fitzgerald, Karam El Bayoumy, Andrea Manni

**Affiliations:** 1 Cancer Prevention Laboratory, Colorado State University, Fort Collins, CO 80523, USA; elizabeth.neil@colostate.edu (E.S.N.); john.mcginley@colostate.edu (J.N.M.); vanessa.fitzgerald@colostate.edu (V.K.F.); 2 College of Medicine, Penn State University, Hershey, PA 17033, USA; kee2@psu.edu (K.E.B.); amani@psu.edu (A.M.)

**Keywords:** breast cancer, cell signaling, docosahexaenoic acid, human cancer cell lines, molecular subtypes, n-3 fatty acids

## Abstract

**Simple Summary:**

Precision onco-nutrition (PON) is the use of specific nutrients and dietary factors to enhance cancer treatment efficacy and to improve the prognosis for long-term survival. PON requires integration of an understanding of nutrient metabolism with knowledge of the signaling pathways characteristic of each molecular subtype of cancer which can be targeted to improve treatment and control efficacies. Herein we report differences among n-3 fatty acids on the growth and survival of established breast cancer cell lines. Docosahexaenoic acid (DHA) is shown to have greater anticancer activity than eicosapentaenoic acid (EPA). Moreover, we show that a penultimate metabolite of one pathway by which DHA is metabolized inhibited the growth of common molecular subtypes of breast cancer via differential effects on identified canonical signaling pathways and the functions within the cell that they regulate. These findings provide a knowledge base for juxtaposing molecular subtype targeted treatment strategies with the adjuvant use of specific n-3 fatty acid metabolites as an example of precision onco-nutrition for the management and control of breast cancer.

**Abstract:**

In vivo evidence of heterogeneous effects of n-3 fatty acids (N3FA) on cell signaling pathways associated with the reduced growth of breast cancer has been reported and is consistent with the expectation that N3FA will not exert uniform effects on all molecular subtypes of the disease. Similarly, available evidence indicates that many metabolites of N3FA are synthesized by mammalian cells and that they exert metabolite-specific biological activities. To begin to unravel the complex relationships among molecular subtypes and effects exerted by specific N3FA metabolites on those pathways, proof-of-concept experiments were conducted using cell lines representative of common molecular subtypes of human breast cancer. N3FA differed in anticancer activity with docosahexaenoic acid (DHA) having greater anticancer activity than eicosapentaenoic acid. 4-oxo-docosahexaenoic (4-oxo-DHA), a penultimate metabolite of 5-lipoxygenase mediated DHA metabolism, induced dose-dependent inhibition of cell number accumulation with apoptosis as a primary effector mechanism. Interrogation of protein expression data using the Ingenuity Pathway Analysis (IPA) bioinformatics platform indicated that 4-oxo-DHA differentially impacted six canonical pathways and the cellular functions they regulate across common molecular subtypes of breast cancer. This included the endocannabinoid pathway for cancer inhibition that has not been previously reported. These findings provide a rationale for juxtaposing molecular subtype targeted treatment strategies with the adjuvant use of specific N3FA metabolites as an example of precision onco-nutrition (PON) for the management and control of breast cancer.

## 1. Introduction

Decades-long efforts to identify individual nutrients and dietary factors associated with differences in risk for cancer have evolved to the elucidation of food patterns associated with changes in cancer risk [1,2,3,4,5]. However, even those efforts fall short if they do not account for impacts of genetically definable differences in metabolism among individuals in the population being investigated as well as differences among cancers associated with organ site and molecular subtype of the disease. Collectively, these circumstances describe a complex array of factors that need to be understood to identify causal relationships linking diet and nutrition to cancer risk. Because of this complexity, there is increasing recognition that a relatively neglected area, i.e., the use of nutrients and dietary factors in combination with the standard of care in cancer treatment, could inform the complex issues involved in cancer risk assessment as described above. Herein we define the use of a specific nutrient metabolite to prime cancer cells to enhance cancer treatment efficacy and improve the prognosis for long-term survival as the field of precision onco-nutrition (PON). However, success depends on ever-increasing knowledge of how individual metabolites of nutrients and other dietary factors exert their effects on signaling pathways that drive cancer progression as well as treatment resistance. 

From the time of the publication of one of the first most comprehensive reviews of diet, nutrition, and cancer, there has been continuous interest in the role of the amount and type of dietary fats in the development of cancer at multiple organ sites [6]. The work reported herein focuses on breast cancer. Of the long string of exciting hypotheses followed by negative clinical trials, the proposed benefits of n-3 fatty acids (N3FA) for breast cancer prevention is a recent example [7,8]. 

By shifting the paradigm in the field of N3FA and breast cancer from risk reduction to PON, i.e., from cancer prevention to cancer control, the existing data can be viewed in a new light. Thus, the evidence that has emerged represents a rich resource for developing anticancer metabolites for use in precision oncology. Those findings include: (1) that parent N3FA, docosahexaenoic acid (DHA), and eicosapentaenoic acid (EPA) are metabolized to active intermediates [9], (2) that canonical and non-canonical pathways are likely to be involved in accounting for the activity of various metabolites [10], and (3) that the heterogeneous responses in cell signaling mediated by N3FA may be due at least in part, to differential sensitivity of breast cancer molecular subtypes to various metabolites of N3FA [10]. In the proof-of-concept work reported herein, an in vitro approach was used to address the complexity represented when differences in N3FA metabolism are juxtaposed with differences among cell lines representative of common molecular subtypes of breast cancer in the signaling pathways that drive cancer growth. In the data-driven series of experiments reported, we first show that of two recognized parent N3FA compounds of dietary origin, DHA has greater anticancer activity against multiple breast cancer cell lines than EPA, thus justifying a focus on this compound. Second, given that multiple metabolites of DHA have been identified, 4-oxo-DHA was used as a metabolic probe to address three questions: (1) is growth inhibited to the same extent in cell lines representative of common breast cancer molecular subtypes, (2) is growth-arrested by the same cellular process(es), and (3) are the same molecular pathways implicated in accounting for anticancer activity across breast cancer cell lines derived from different molecular subtypes of the disease?

## 2. Materials and Methods

### 2.1. Biological Reagents and Chemicals 

Human breast cancer cell lines of BT474, MCF7, SKBR3, MDAMB231, and MDAMB468 were obtained from American Type Culture Collection (Manassas, VA, USA). Primary antibodies used in this study have previously been reported by us for use in Western blot probing of both rat mammary gland and mammary tumors as well as human breast cancer cell lines [10]. Table S1 and Figure S1, respectively, provide a detailed list of the vendors and catalog numbers of the antibodies that were used and electropherogram profiles illustrating the specificity of those antibodies. Dulbecco′s modified Eagle′s medium Ham′s F12 50/50 mix with L-glutamine (DMEM/F12) and fetal bovine serum (FBS) were purchased from Invitrogen Corp. (Carlsbad, CA, USA). 

Docosahexaenoic acid (DHA), eicosapentaenoic acid (EPA), and linoleic acid (LA) were purchased from Sigma Aldrich (St. Louis, MO, USA). 4-oxo-docosahexaenoic was synthesized as previously reported [11,12]. Prior to treating cells in culture each of the compounds were complexed to bovine serum albumin (BSA) to reduce toxicity associated with the detergent effects of free fatty acids. FA-free BSA solution in plain cell culture medium was used and 0.05 M Na_2_CO_3_ was added to DHA, 4-oxo-DHA, EPA, or LA at 5 mg DHA/mL 0.05 M Na_2_CO_3_. The vials were flushed with N_2_ and then vortexed extensively. The materials were calculated to make 2.5 mM FA-BSA as a complex at the FA:BSA = 3:1 mole of ratio was needed. DHA (MW:328.5) of 5 mg in 1 mL 0.05 M Na_2_CO_3_, BSA (MW:68,000):15% Solution. The FA-BSA complex was sterile filtered under a sterile cell culture hood, aliquoted, and stored at −80 °C.

### 2.2. Cell Culture and Rationale

Five human breast cancer cell lines were used in this study: BT474, MCF7, SKBR3, MDAMB231, and MDAMB468. They represent 5 subtypes: luminal A (MCF7), luminal B (BT474), post-EMT (mesenchymal transition) (MDAMB231), human epithelial growth factor receptor 2 (HER2) over-expressing (SKBR3), and basal-like (MDAMB468). Basal-like breast carcinomas are characterized by high expression of basal cyto-keratins, low or absent expression of estrogen receptor, progesterone receptor, and HER2/neu, and expression of epidermal growth factor receptor (EGFR). MDAMB231 and MDAMB468 are classified as triple-negative/basal-like although MDAMB231 has a mesenchymal-like phenotype.

### 2.3. Assessment of Cell Number Accumulation 

Cell lines were grown at 37 °C in a humidified incubator containing 5% CO_2_ in DMEM and F-12 medium (Mediatech, Inc., Manassas, VA, USA) (1:1 ratio) containing 10% fetal bovine serum (HyClone, Cytiva, Marlborough, MA, USA). The next day, cells were provided fresh medium at doses of 0, 25, 50, 100, and 150 µM with DHA, 4-oxo-DHA, LA, or EPA. On days 1, 3, and 5 after exposure, cells were fixed with 1% glutaraldehyde, replaced with phosphate-buffered saline (PBS), and stored at 4 °C. At the end of an experiment, all the plates were stained with 0.02% aqueous crystal violet for 30 min and rinsed with deionized water. After re-dissolving the bound crystal violet in 70% ethanol, the absorbance was determined at 590 nm using a Spectromax Plus Microplate Spectrophotometer System (Molecular Devices, Sunnyvale, CA, USA).

### 2.4. Assessment of Cell Proliferation and Apoptosis

#### 2.4.1. Cell Proliferation Assay

Cells (*n* = 6000) were suspended with 100 uL of DMEM and F-12 medium (1:1) ratio containing 10% fetal bovine serum and seeded into flat-bottomed 96 wells. Twenty-four hours after initial seeding, cells were allowed to grow in either the control medium or the same medium supplemented with 25 μM 4-oxo-DHA for 3 days in a 5% CO_2_ incubator at 37 °C. This dose of 4-oxo-DHA was chosen because it induced a similar degree of inhibition of cell number accumulation across these three cell lines after 3 days of treatment. The cell proliferation was determined by a proliferation assay kit according to the standard manufacturer’s protocol (MBL International Corporation). Absorbance was measured at a 450-nm wavelength with a reference wavelength of 650 nm using a SpectraMax M5 Microplate Reader (Molecular Devices, Sunnyvale, CA, USA). The experiments were performed in seven replicates and repeated three times. 

#### 2.4.2. Determination of Apoptosis 

The induction of apoptosis in cultured cells was determined morphologically by fluorescent microscopy after labeling with acridine orange and ethidium bromide, as described by Duke and Cohen [13]. Floating cells and enzymatically dissociated adherent cells were pooled and washed three times in PBS. The cells were centrifuged at 300× *g*, and the pellet was gently resuspended in the culture media to make a suspension containing 1 × 10^6^ cells/mL. A 25-mL-aliquot of the cell suspension was mixed with 1 mL of a dye solution containing 100 mg/mL acridine orange and 100 mg/mL ethidium bromide prepared in PBS. This mixture was placed on a slide, topped with a coverslip, and examined under a 40× objective using a microscope equipped with epi-illumination and a fluorescein filter set (Axioskop 2, Carl Zeiss, Inc., White Plains, NY, USA). To minimize subjective bias, the analyst was blinded to the identity of the treatment sample. At least 200 cells were evaluated per sample. Live and dead apoptotic cells were identified by nuclear condensation of chromatin stained by acridine orange and ethidium bromide, respectively. The quantification of apoptotic cells was calculated as a percentage of the total number of cells counted. The experiments were performed in triplicate and repeated three times.

### 2.5. Western Blot Analyses 

Three cell lines, BT474 (Luminal B), SKBK3 (Her2 over-expression), MDAMB468 (TN) were treated with 4-oxo-DHA at 25 µm for 3 days and lysed at 4 °C in the lysis buffer (40 mM Tris-HCl [pH 7.5], 0.1% Triton X-100, 0.25 M sucrose, 3 mM EGTA, 3 mM EDTA, 50 μM β-mercaptoethanol, 1 mM phenyl-methylsulfonyl fluoride, and complete protease inhibitor cocktail (Calbiochem, San Diego, CA, USA). The lysates were centrifuged at 7500× *g* for 10 min at 4 °C and supernatant fractions were collected and stored at −80 °C. Supernatant protein concentrations were determined by the Bio-Rad protein assay (Bio-Rad, Hercules, CA, USA). Western blotting was performed as described previously [14]. Briefly, 40 μg of protein lysate per sample were subjected to 8–16% sodium dodecyl sulfate-polyacrylamide gradient gel electrophoresis (SDS-PAGE) after being denatured by boiling with the SDS sample buffer (63 mM Tris-HCl [pH 6.8], 2% SDS, 10% glycerol, 50 mM DTT (dithiothreitol), and 0.01% bromophenol blue) for 5 min. After electrophoresis, proteins were transferred to a nitrocellulose membrane. The levels of protein expression were determined using specific primary antibodies, followed by treatment with the appropriate peroxidase-conjugated secondary antibodies, and visualized by LumiGLO reagent Western blotting detection system. The chemiluminescence signal was captured using a ChemiDoc densitometer (Bio-Rad, Hercules, CA, USA) that was equipped with a CCD (charged-coupled device) camera having a resolution of 1300 × 1030. Quantity One software (Bio-Rad, Hercules, CA, USA) was used in the analysis of the actin-normalized scanning density data.

### 2.6. Statistical Analyses

For statistical analysis of data resulting from Western blotting, the actin-normalized scanning density data obtained from the ChemiDoc scanner using Quantity One (Bio-Rad) were first rank transformed. This approach is particularly suitable for semiquantitative measurements that are collected as continuously distributed data, as is the case with Western blots. The ranked data were then subjected to the non-parametric Kruskal Wallis rank test. Ratio data were computed from the scanning units derived from the densitometric analysis, i.e., the arbitrary units of optical density for variables stated, and then the ratios were rank transformed and similarly evaluated. All analyses were performed using Systat statistical analysis software, version 13.

### 2.7. OPLS-DA 

As an independent approach to validating which proteins may mediate the biological effects of 4-oxo-DHA, we used orthogonal projections to latent structures for discriminant analysis (OPLS-DA), a supervised, class-based method where class membership is assigned to samples and used to elicit maximum data separation. Visualization of OPLS-DA Scatter plots of the first two-score vectors for each model were drawn based on Hotelling’s multivariate T2, to identify outliers that might bias the results of OPLS-DA. For OPLS-DA, class separation was shown as the first predictive score plotted against the first orthogonal score to visualize the within- and between-class variability associated with the first principal component. S-plots were constructed to identify influential proteins in the separation of breast cancer cell lines. S-plots based on the first principal component show reliability (modeled correlation) plotted against feature magnitude (loadings or modeled covariance). If proteins have variation in correlation and covariance between classes, this plot will assume an S-shape (giving the plot its name), with heavily influential features separating from other features at the upper right and lower left tails of the feature cloud within the model space. Only proteins influential in distinguishing among breast cancer cell lines are reported. All analyses were done using SIMCA-P+ v.12.0.1 (Umetrics, Umea, Sweden). 

### 2.8. Bioinformatic Analyses

For bioinformatic analyses, samples were grouped by experimental conditions, and protein expression data was normalized and log-transformed. Statistical analysis was performed to generate non-phosphorylated, phosphorylated, and ratio (phosphorylated to total protein) expression values each with associated *p*-value and FDR value. The resulting data were exported to an Excel file and uploaded to IPA (Qiagen Digital Insights, Redwood City, CA, USA) for further analysis. Phosphorylation Core Analysis was performed in Ingenuity Pathway Analysis (version 68752261) for each cell line. The canonical pathways feature was utilized and a z-score ≥ 2 was the cutoff for a statistically significant activated pathway while z ≤ 2 was the cutoff for a statistically significant inhibited molecule. Comparison analysis across cell lines was used to show predicted activation or inhibition of diseases and biological functions based on z-score. The analysis match feature was used to query potential drug treatments best predicted to work in concert with DHA treatment.

## 3. Results

### 3.1. DHA Is Superior to EPA in Inhibiting Cancer Cell Growth

**Figure 1:**
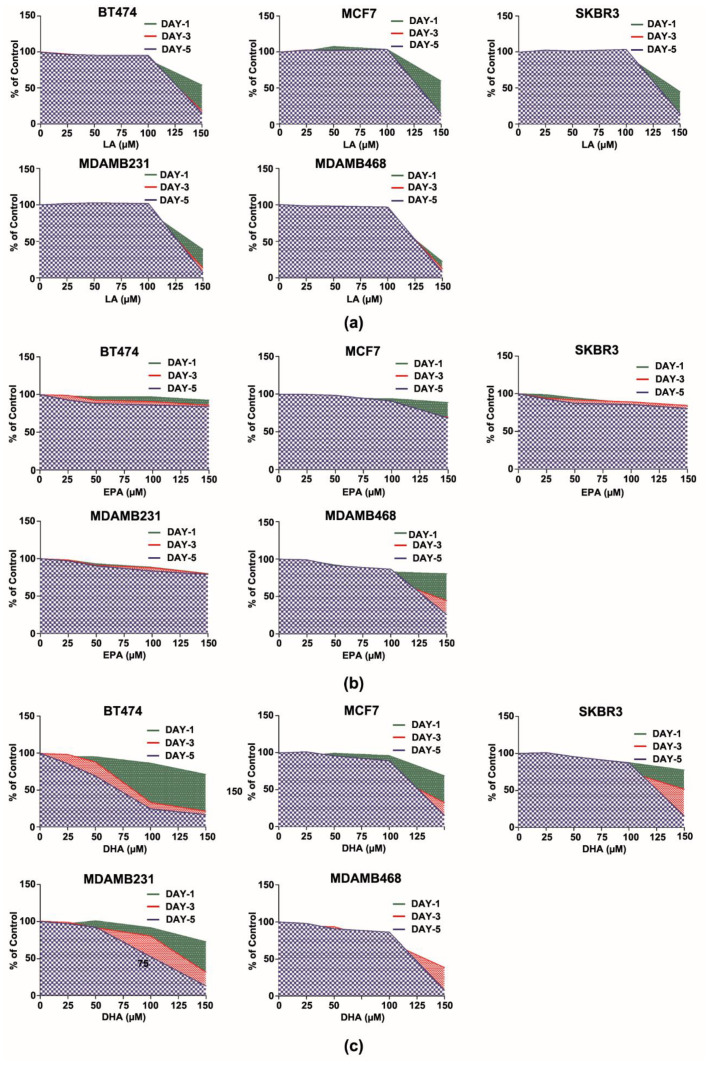
Dose- and time-dependent effects of n6- or n3-fatty acids on the growth of five subtype human breast cancer cells (BT474, MCF7, SKBR3, MDAMB231, and MDAMB468) graphed using the area under the curve (AUC) as a percent of the untreated control. (**a**) Linoleic acid (LA, n6); (**b**) Eicosapentaenoic acid (EPA, n3); (**c**) Docosahexaenoic acid (DHA, n3).

Breast cancer cells representative of prevalent molecular subtypes were used to determine the comparative effects of DHA and EPA on cell number accumulation, that is the net effect of proliferation and death on cell number as a function of concentration and time of exposure. Linoleic acid (LA), an n-6 fatty acid, was used as a negative control and all fatty acids were bound to bovine serum albumin to avoid nonspecific detergent effects of free fatty acids. To facilitate the integration of a large amount of data and to permit the visualization of the effects of fatty acid dose and duration of exposure on the kinetics of cell number accumulation, values were computed as a percent of control and were graphed using an area under the curve (AUC) function. LA had little effect on cell number accumulation; however, at concentrations above 100 µM, cell number decreased rapidly, most likely because of nonspecific toxicity due to increased levels of free fatty acids being released from albumin into the media (Figure 1a). Effects of EPA (Figure 1b) were similar to those of LA, although evidence of small inhibitory effects was observed on cell number accumulation at concentrations ≤ 100 µM, particularly for BT474, SKBR3, and MDAMB231 (*p* < 0.05). On the other hand, DHA showed the greatest inhibitory effects at concentrations ≤ 100 µM with dominant effects at 3 and 5 days of exposure, primarily in BT474 and MDAMB231 (*p* < 0.01). These data provided a rationale for focusing on DHA.

### 3.2. 4-oxo-DHA Exerts Dominant Effects on HER-2/Neu Negative Molecular Subtypes

**Figure 2:**
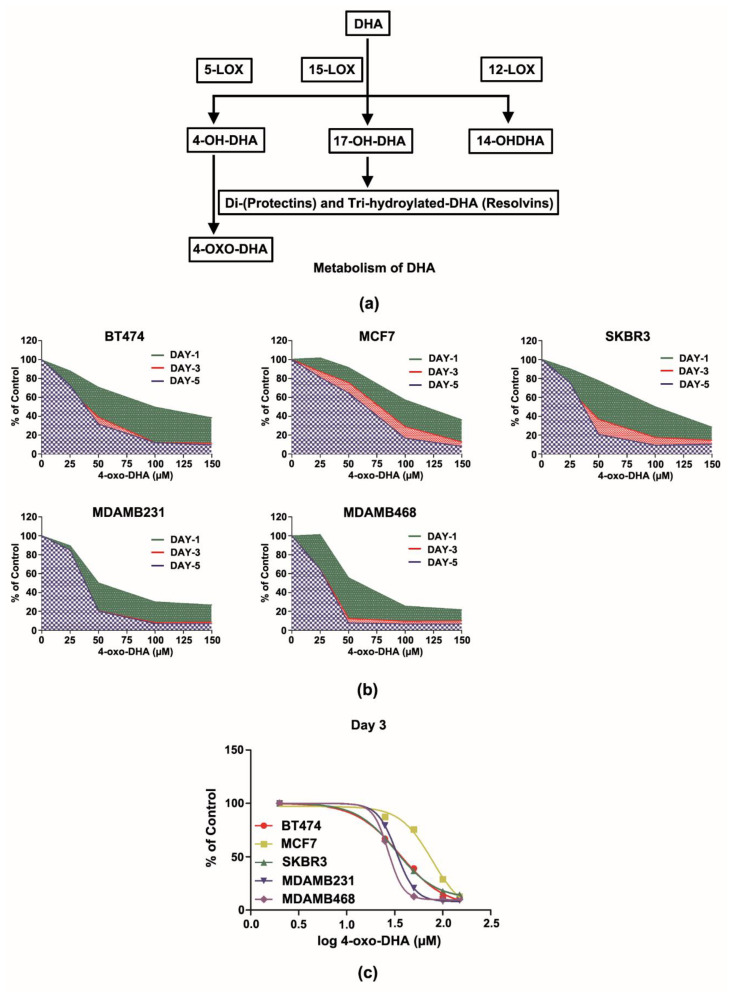
Metabolism of docosahexaenoic acid (DHA) and the dose- and time-dependent effects of 4-oxo-DHA on the growth of five subtype human breast cancer cells (BT474, MCF7, SKBR3, MDAMB231, and MDAMB468) graphed using an area under the curve (AUC) or the half-maximal inhibitory concentration (IC_50_) as a percent of untreated control. (**a**) Metabolism of DHA; (**b**) an area under the curve analysis; (**c**) the half-maximal inhibitory concentration for 3 days of treatment.

DHA is known to be further metabolized within the cell by 5, 12, and 15- lipoxygenases to a number of chemical species (Figure 2). As we have observed elevated concentrations of 4-OH-DHA in the plasma and tissue, as well as 4-oxo-DHA in plasma of DHA, treated animals [11,15,16,17], the decision was made to focus on the pathway catalyzed by the 5-lipoxygenase and that ultimately yields 4-oxo-DHA. Since our goal was to minimize the potential impact of differences among cell types in enzymatic activity relating to metabolite synthesis, 4-oxo-DHA was used as a metabolic probe for these studies. At the same concentrations and durations of exposure that were used to evaluate the parent DHA, significant dose and time-dependent reductions were observed in cell number accumulation across all cell lines exposed to 4-oxo-DHA, *p* < 0.001 (Figure 2). However, there were differences among cell lines in responsiveness. Growth inhibition ranged from as little as 25% in the MCF7 cell line to 87% in the MDAMB468 cell line when the cells were exposed to 50 µM 4-oxo-DHA for 3 days. These data are clearly consistent with the hypothesis that different molecular subtypes of breast cancer respond differently to the same DHA metabolite. The growth patterns also differed over time as seen using AUC analyses. To further quantify this effect, the same data were used for IC_50_ analysis. The IC_50_ analysis for Day 3 across the 5 cell lines (Figure 2) and the IC_50_ analyses for days 1 and 5 (Figure S2) indicated that the effects of 4-oxo-DHA are not immediate and that they are accelerated as the exposure dose is increased. In performing the IC_50_ analysis on day 3, it was observed that the concentration was similar for BT474, SKBR3, and MDAMB468. As these cell lines represent prevalent molecular types of breast cancer, they were the focus of subsequent studies. 

### 3.3. 4-oxo-DHA Inhibits Cell Proliferation and Induces Apoptosis

Under dose and exposure conditions of 4-oxo-DHA that reduce cell number accumulation by 50% (25µM for 3 days) in BT474, SKBR3, and MDAMB468 cells, indicators of cell proliferation and apoptosis were assessed, and cell lysates were Western blotted for proteins involved in the regulation of these processes. For assessment, the results of 8 replicate, independent experiments for each cell line at 0 or 25 µM 4-oxo-DHA were evaluated statistically. Rates of apoptosis induction (apoptosis index, %) and suppression of cell proliferation (MTT index, OD) were statistically different across cell lines, although this “snapshot in time” needs to be interpreted with caution since there are recognized biases in these estimates. To complement these indices, the levels of proteins involved in these processes was determined by Western blotting (Table 1). Specifically, key proteins that regulate the G_1_–S transition in the cell cycle and the induction of apoptosis were assessed. The levels of cyclin-D1 and phospho-Rb were reduced and levels of 2 cyclin-dependent kinase inhibitors, p21, and p27 were elevated in 4-oxo-DHA treated cells. Relative to apoptosis and consistent with the elevated apoptotic index observed in 4-oxo-DHA treated cells, the level of Apaf-1 and the Bax/BCL2 ratio were elevated; whereas, the level of cleaved PARP (PARP89/116 ratio) was minimally affected. Collectively these data are consistent with the induction of apoptosis via the intrinsic pathway although they suggest that the magnitude of induction may be overestimated by the apoptotic index. 

**Figure 3:**
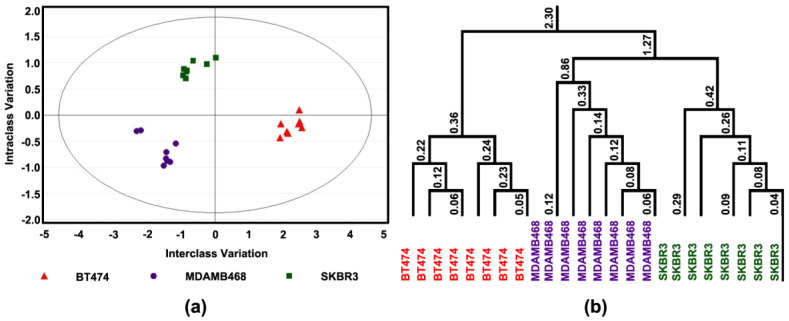
Effect of 4-oxo-DHA on Cell Proliferation and Apoptotic Cell Death. Levels of cell proliferation or apoptosis and associated target proteins in three human breast cancer cell lines (BT474, SKBR3, and MDAMB468); (**a**) Orthogonal projections to latent structures-discriminant analysis (OPLS-DA) shows a 3-class supervised model and partition the sources of variation; (**b**) To visualize the misclassification rate, the dendrogram depicts hierarchical clustering patterns among three different cell lines.

These data were subjected to advanced multivariate regression techniques using orthogonal projections to latent structures discriminant analysis (OPLS-DA) to determine if these differences were sufficient to distinguish among cell lines; complete separation was observed (Figure 3a). The apoptosis/proliferation response was most distinctive in BT474 (triple positive) and the responses of MDAMB468 (triple-negative) and SKBR3 were more like one another (Figure 3b). 

Collectively, these analyses indicated that apoptosis induction is the driver accounting for inhibition of cell number accumulation across cell lines. A key question emerged during these analyses: were differences in response to 4-oxo-DHA due to distinctive patterns of deregulated cell signaling characteristic of each cell line? To pursue this question, experiments were extended to signaling pathways affected by PPARγ activation and to pathways that have direct effects on cell survival mediated via the mTOR regulatory network based on work reported in [10].

### 3.4. 4-oxo-DHA Induces the Molecular Signature Indicative of PPARγ Activation

Given that a considerable body of evidence indicates that N3FA mediate effects on cell signaling via binding to PPARα, β/δ, and γ, Western blot analyses were conducted to determine levels of PPAR β/δ, and γ. PPARα was not measured since it is primarily expressed in hepatic tissue (Table 2). PPARγ content was increased across cell lines by 4-oxo-DHA; whereas, the effect on β/δ was variable. Another family of cell surface receptors that can activate gene expression following fatty acid-binding are G-protein–coupled receptors. One such protein, GRP120, is generally associated with macrophages, which was of interest as macrophage invasion of tumors is common. The level of GRP120 was elevated in BT474 and MDAMB468 but unaffected in SKBR3, a pattern of induction across cell lines that differed from that of PPAR β/δ. Because N3FA has also been reported to affect transcriptional activity related to inflammation, intermediary metabolism, and cell fate, effects on additional transcription factors were assessed, that is, the content of phospho-NF-κB p65^Ser536^, phospho-FOXO-3a^Thr32^, Hif-1α, and SIRT-1. As shown in Table 2, NF-κβ p65^Ser536^, Hif-1α, and GADD153 were lower in BT474 and MDAMB468 but unaffected in SKBR3; whereas, SIRT1 was only affected (reduced) in BT474. 

**Figure 4:**
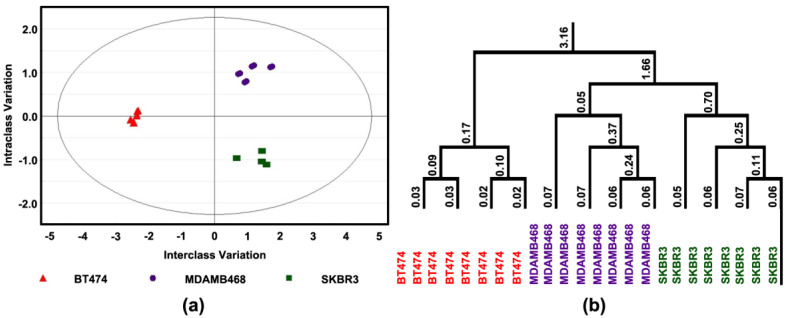
Effect of 4-oxo-DHA on Cell Transcription Factors. Levels of cell proliferation or apoptosis and associated target proteins in three human breast cancer cell lines (BT474, SKBR3, and MDAMB468); (**a**) orthogonal projections to latent structures-discriminant analysis (OPLS-DA) shows a 3-class supervised model and partition the sources of variation; (**b**) to visualize the misclassification rate, the dendrogram depicts hierarchical clustering patterns among three different cell lines.

In addition, the same approach as described above was used to determine if these differences were sufficient to distinguish among cell lines; complete separation was observed (Figure 4a). The response was most distinctive in BT474 (triple positive) and the responses of MDAMB468 (triple-negative) and SKBR3 were more like one another (Figure 4b).

### 3.5. 4-oxo-DHA Induces Effects on Activity of Proteins in the mTOR Regulatory Network

**Figure 5:**
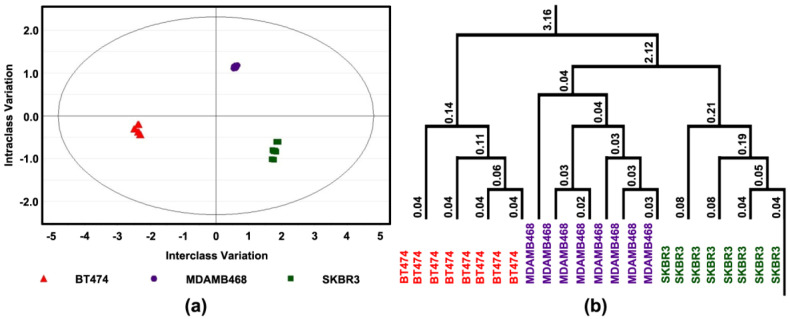
Effect of 4-oxo-DHA on Growth Factor Signaling. Levels of cell proliferation or apoptosis and associated target proteins in three human breast cancer cell lines (BT474, SKBR3, and MDAMB468); (**a**) Orthogonal projections to latent structures-discriminant analysis (OPLS-DA) shows a 3-class supervised model and partition the sources of variation; (**b**) To visualize the misclassification rate, the dendrogram depicts hierarchical clustering patterns among three different cell lines.

The evaluation of insulin-related signaling involved a large number of proteins in the Akt-mTOR-AMPK–signaling network. A summary of the levels of expression for protein components of the mTOR network (IGF-1R and PI3Kp110) and the activity of key regulatory nodes in the mTOR signaling, measured as the ratio of the phospho-specific residue to total protein is provided (Table 3). The responses among the 3 cell lines were distinct (Figure 5a). The response was most distinctive in BT474 (triple positive) and the responses of MDAMB468 (triple-negative) and SKBR3 were more like one another (Figure 5b). 

### 3.6. Lipid Synthesis Appears to Be a Nexus for cross Talk among Pathways

The induction of PPARγ activity and suppression of mTOR activity are expected to modulate de novo lipid biosynthesis which is considered a prerequisite for cell growth. To assess the effects of 4-oxo-DHA on lipid biosynthetic potential, cell lysates were blotted for levels of proteins involved in regulating lipid biosynthesis. The effects of 4-oxo-DHA on these proteins and the activity of acetyl-CoA carboxylation, a key control point in fatty acid synthesis is shown for each cell line (Table 4). The level of phosphorylated ACC was increased by 4-oxo-DHA, consistent with decreased activity of the enzyme and reduced lipid biosynthesis. However, the effects of 4-oxo-DHA on other proteins involved in lipid synthesis, i.e., FASN, HMGCR, and SREBP-1 were variable across cell lines. As work in this field progresses, these findings indicate the value of measures of enzyme activity of proteins involved in lipid synthesis and of chromatographic analyses of tissue lipid profiles to better understand how alterations in lipid metabolism can be leveraged for PON. 

### 3.7. Interrogation of Protein Expression Data Using the Ingenuity Pathway Analysis (IPA) Bioinformatics Platform

**Figure 6:**
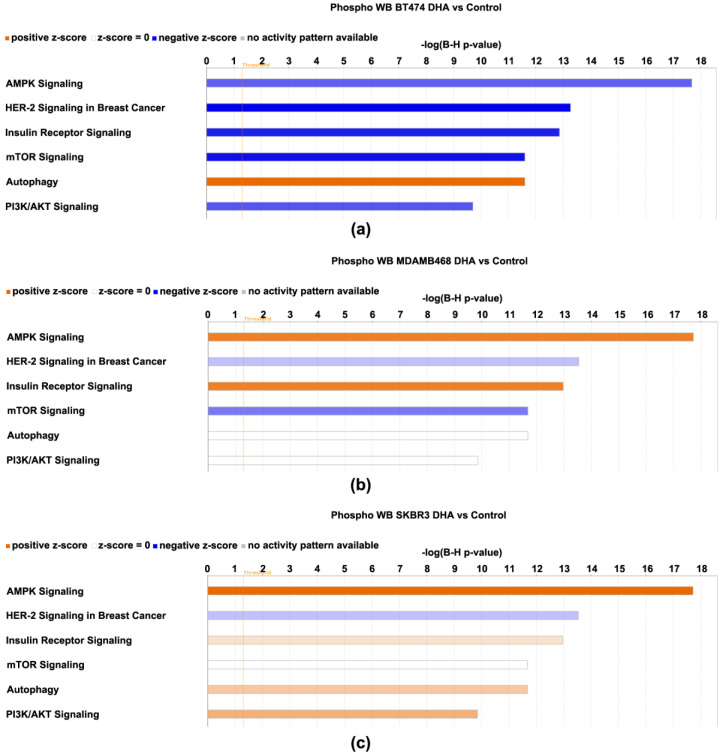
Canonical pathways affected by 4-oxo-DHA as predicted by the phosphoproteome data sets listed in Table 1, Table 2, Table 3 and Table 4. Canonical pathways shown for cell line (**a**) BT474, (**b**) MDAMB468, and (**c**) SKBR3. The −log(B-H *p*-values) is the multi = comparison-adjusted probability that the association between the protein expression set and the canonical pathway is due to chance. The direction of the differences in expression between the treatment and the control for each protein component was compared to that tabulated in the IPA knowledge basis (>80,000 database entries) that support the canonical pathways that have been annotated and a z-score was computed. A z-score ≤ −2 indicates the pathway is inhibited and ≥2 that the pathway is activated. If the z-score is between −2 and 2, no prediction of inhibition or activation is deduced. Shades of red indicate that the pathway was activated by treatment with 4-oxo-DHA; greater color intensity indicates stronger evidence of activation. Shades of blue indicate that the pathway was inhibited by treatment with 4-oxo-DHA; greater color intensity indicates stronger evidence of inhibition. The absence of coloration indicates that the evidence was not strong enough to permit a prediction of pathway status.

**Figure 7:**
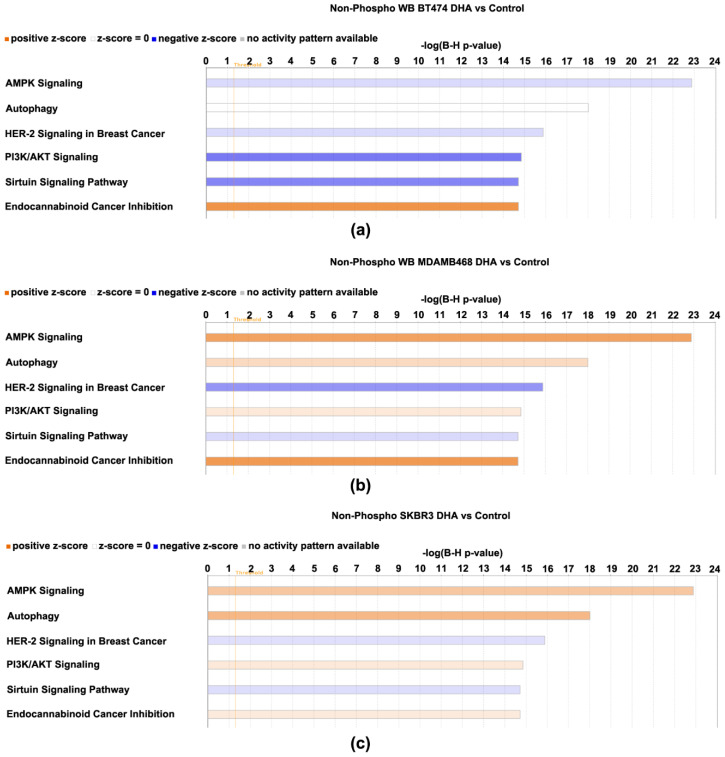
Canonical pathways affected by 4-oxo-DHA across three molecular subtypes of breast cancer cell lines. Canonical pathways affected by 4-oxo-DHA as predicted by the nonphosphorylated proteome data sets listed in Table 1, Table 2, Table 3 and Table 4. Canonical pathways shown for cell line (**a**) BT474, (**b**) MDAMB468, and (**c**) SKBR3.The −log(B-H *p*-values) is the multicomparison adjusted probability that the association between the protein expression set and the canonical pathway is due to chance. The direction of the differences in expression between the treatment and the control for each protein component was compared to that tabulated in the IPA knowledge basis (>80,000 database entries) that support the canonical pathways that have been annotated and a z-score was computed. A z-score ≤ −2 indicates the pathway is inhibited and ≥2 that the pathway is activated. If the z-score is between −2 and 2, no prediction of inhibition or activation is deduced. Shades of red indicate that the pathway was activated by treatment with 4-oxo-DHA; greater color intensity indicates stronger evidence of activation. Shades of blue indicate that the pathway was inhibited by treatment with 4-oxo-DHA; greater color intensity indicates stronger evidence of inhibition. The absence of coloration indicates that the evidence was not strong enough to permit a prediction of pathway status.

All protein expression data, i.e., both phosphorylated and non-phosphorylated, were subjected to log2-fold change computational analysis (4-oxo-DHA vs. control) and statistical analysis with false discovery rate (FDR) correction. Those data were evaluated using the IPA Core Analysis algorithm. The components of the protein expression panel were matched for their overlap with the >250 canonical pathways that are annotated in IPA using a Fisher Exact Test with FDR (B-H) correction for multiple comparisons. The smaller the *p*-value, the lower the probability that the association between the protein expression set and the canonical pathway is due to chance. In addition, the direction of the differences in expression between the treatment and the control (magnitude and direction) for each protein component was compared to that tabulated in the IPA knowledge basis (>80,000 database entries) that support the canonical pathways that have been annotated and a z-score was computed. A z-score ≤ 2 indicates the pathway is inhibited and ≥2 that the pathway is activated. If the z-score is between −2 and 2, no prediction of inhibition or activation is deduced. The results of the analysis are shown in Figure 6 and Figure 7 and Figure S3. The top 6 canonical pathways identified using the phospho-proteome data are shown (Figure 6) in addition to the top 6 pathways identified using the non-phosphorylated protein expression analysis (Figure 7). 

The phospho-proteome data identifies the mTOR signaling network, which integrates signaling effects of AMPK and PI-3 kinase/AKT on mTOR activity as being differentially regulated across BT474, SKBR3, and MDAMB468 even though 4-oxo-DHA suppresses cell number accumulation to a similar extent at 25 µM for 3 days of exposure. Also, a predicted induction of autophagy was observed that also varied in its intensity across cell lines. The non-phosphorylated protein data resulted in the identification of two additional canonical pathways, sirtuin signaling (inhibited) and endocannabinoid signaling associated with cancer inhibition (activated). HER-2 signaling in breast cancer was identified as being inhibited in all analyses in which a prediction was made. 

#### Regulation of Diseases and Cell Functions

**Figure 8:**
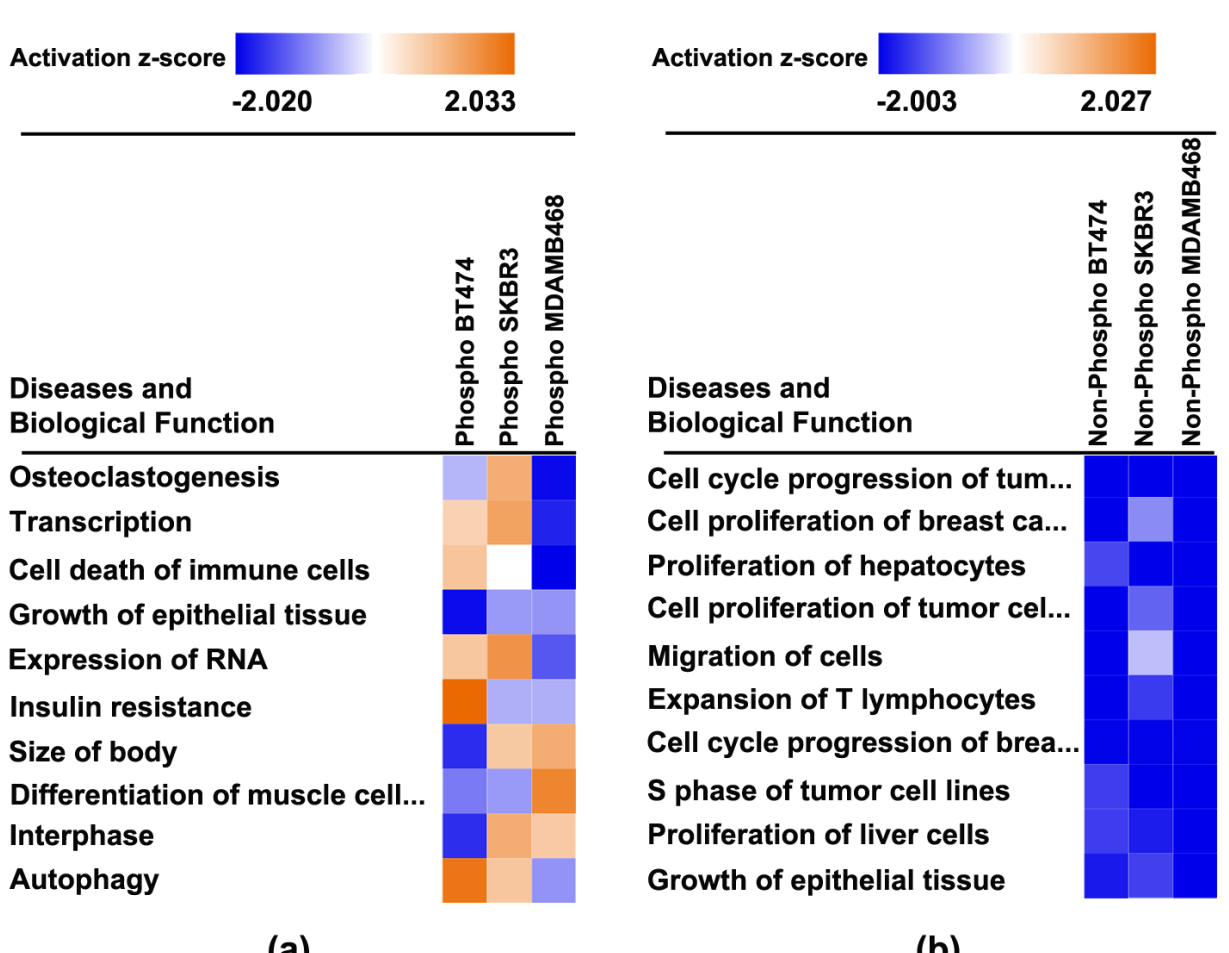
Regulation of diseases and cell functions by 4-oxo-DHA was observed across three molecular subtypes of breast cancer cell lines. The Core Analysis algorithm in IPA also identifies diseases and functions within cells that are consistent with the protein expression data. Those data are shown in heat maps based on the expression of (**a**) phosphorylated and (**b**) non-phosphorylated proteins. Shades of red indicate that the biological function was activated by treatment with 4-oxo-DHA; greater color intensity indicates stronger evidence of activation. Shades of blue indicate that the biological function was inhibited by treatment with 4-oxo-DHA; greater color intensity indicates stronger evidence of inhibition. The absence of coloration indicates that the evidence was not strong enough to permit a prediction of biological function status.

The Core Analysis algorithm in IPA also identifies diseases and functions within cells that are consistent with the protein expression data. Those data are shown in heat maps based on the expression of non-phosphorylated and phosphorylated proteins (Figure 8). This data is also detailed in Table S2.

Effects are variable across cell lines. The prediction of effects on cell proliferation (decreased), cell cycle progression (decreased), apoptosis induction, and lipid metabolism are variable but consistent with the data reported (Table 1, Table 2, Table 3 and Table 4). More importantly, insights about other diseases and cellular functions that may be affected by 4-oxo-DHA treatment in vivo are noted. Of particular interest are the effects on components of the immune response which may offer novel opportunities for affecting breast cancer treatment and disease recurrence. To our knowledge, a role for immune modulation has not been previously reported.

### 3.8. Regulation of mTOR Signaling 

**Figure 9:**
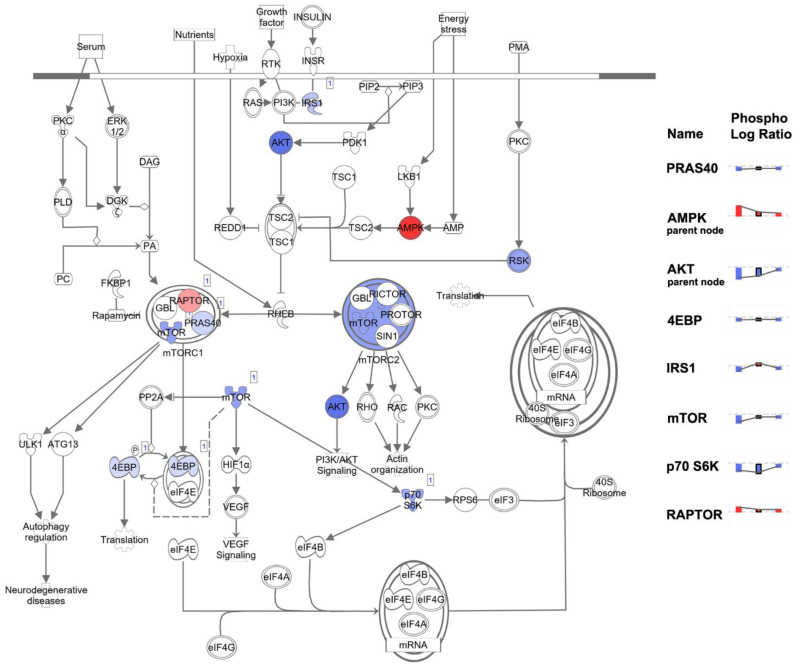
Effect of 4-oxo-DHA on the mTOR signaling pathway was diagrammed in IPA and corresponding expression data from the BT474 cell line was overlaid on the pathway. Overlaid expression is shown as the ratio of phosphorylated to total protein. The chart on the right shows the expression of major regulatory nodes of the mTOR signaling pathway while illustrating a pattern of differential regulation across the cell lines BT474, SRBR3, and MDAMB464, respectively. Red indicates protein activation and blue indicates inhibition.

Given the central role of the mTOR signaling network and its differential regulation by cell type in accounting for effects of 4-oxo-DHA as reported (Figure 6 and Figure 7), that pathway was diagrammed in IPA and corresponding expression data were overlaid on the pathway to illustrate differential regulation across cell lines (Figure 9). The node charts on the figure reveal these differences at primary regulatory nodes of the pathway. 

### 3.9. Analysis Match for Overlap of the 4-oxo-DHA Mediated Signaling in MDAMB-468 with Breast Cancer Treatment Data Sets

The Analysis Match algorithm in IPA was used to identify published databases from the evaluation of breast cancer cell lines with targeted chemotherapeutics that induced a pattern of expression like that induced by 4-oxo-DHA. Analysis Match compared the pattern of protein expression in the MDAMB468 breast cancer cell line, which is representative of a poor prognosis molecular subtype of breast cancer, with more than 100,000 highly curated and quality-controlled human, mouse, and rat disease and oncology datasets re-processed from SRA, GEO, Array Express, TCGA, and LINCS. This “analysis-to-analysis” matching is based on shared patterns of Canonical Pathways, Upstream Regulators, Causal Networks, and Diseases and Functions. IPA computed a z-score for the match of the “query” signature against the signatures of all other analyses. Table 5 shows top matches ranked by z-score, and the analysis is further detailed in Table S3. This illustrates how hypothesis-driven PON can be developed and advanced to evaluation using co-clinical translational approaches.

## 4. Discussion

There are many examples in the literature documenting how a detailed understanding of the chemical species produced during the metabolism of a nutrient or dietary factor within mammalian systems has led to the development of pharmaceuticals that specifically target a cellular function and/or disease process. Prominent examples include natural and synthetic metabolites of vitamin A and vitamin D, but many other examples exist [18,19]. Because of methodological obstacles and the complexity of their metabolism, the application of metabolite-specific strategies to harness the activity of over 100 bioactive metabolites produced during fatty acid metabolism has lagged other categories of nutrients/dietary factors in being exploited for therapeutic benefit. However, technical advances now make it possible to systematically evaluate which fatty acid metabolites have the potential to improve cancer treatment [20]. Of the various categories of fatty acids, N3FA appears to exert effects on many of the deregulated cellular and molecular processes identified as hallmarks of cancer [21]. Proof-of-concept evidence presented herein supports the premise that N3FA metabolites could be used as well-tolerated oral adjuvants that improve treatment efficacy and long-term survival as an example of PON. 

There is a significant precedent for considering dietary N3FA as an inseparable mixture consisting primarily of EPA and DHA and this paradigm is reflected in the formulation of Lovaza^®^, a drug used in the treatment of disorders in lipid metabolism including cardiovascular disease. However, EPA and DHA are now commercially available as single-agent formulations. Accordingly, the data reported indicate that DHA (Figure 1c) inhibits breast cancer cell growth in comparison to EPA or linoleic acid (Figure 1a,b), that these effects are achievable at concentrations of DHA that can be obtained with the use of fish oil supplements, and that the effects of DHA vary among cell lines derived from common molecular subtypes of breast cancer. These in vitro findings regarding the greater efficacy of DHA relative to EPA are consistent with previous reports. The concentrations of DHA and EPA used in these cell culture experiments are lower than the plasma concentrations detected in women at risk for breast cancer that were given 4g of Lovaza^®^ daily for 6 months [22]. They are also lower concentrations than reported in other investigations [15,23,24] and provided the rationale for our decision to focus on a metabolite of DHA. Higher concentrations were not investigated because we observed evidence of non-specific toxicity at exposure concentrations ≥100 µM of fatty acids even though they were bound to serum albumin before in vitro treatment was initiated. We speculate that toxicity was due to the well-known detergent effect of free fatty acids on cells and resulting membrane leakage and necrotic cell death. Relative to the differential effects of N3FA on molecular subtypes, we are unaware of any previous reports of evaluation of N3FA on a panel of breast cancer molecular subtypes with the expressed purpose of a comparative analysis on cell accumulation kinetics. Our finding of differential sensitivity is consistent with our recent report in vivo where we speculated that heterogeneity of molecular signaling responses in mammary carcinomas might be due to differential effects associated with molecular subtypes [10] and this could explain at least in part the inconsistencies observed in both preclinical and clinical investigation of N3FA in the prevention of breast cancer [8].

The second series of experiments reported herein focused on a specific metabolite of DHA in vivo following the initial hydroxylation of DHA by lipoxygase-5. 4-OH-DHA has been detected in the plasma of women taking Lovaza, orally, and also in rats given cancer-preventive concentrations of dietary DHA [15,23,24]. Initial work with 4-oxo-DHA (Figure 2) established that like the parent compound DHA, the 4-oxo-DHA metabolite inhibited cell number accumulation, that the effect was dose-dependent, and that the degree of inhibition varied over time and by molecular subtype with the greatest effects being on the MDAMB cell lines that are triple-negative breast cancer cells considered to be basal cell type in their origin. At this time, we have no basis to speculate on a mechanism for selective effects of an N3FA metabolite on mammary epithelial cells of basal versus luminal origin. 

For subsequent experiments, three cell lines were chosen for more detailed analyses. The cell lines selected represent prevalent types of breast cancer, i.e., steroid hormone receptor-positive disease that overexpresses Her2/Neu (BT474) and two steroid hormone receptor-negative molecular subtypes that vary in Her2/Neu expression status. SKBR3 over-expresses Her2/Neu; whereas, MDAMB468 is a triple-negative cell line. Both molecular subtypes have a poor prognosis. The experiments were performed on cells after three days of exposure to 4-oxo-DHA at a dose of 25 µM, which inhibited growth by 50 percent (Figure 3, Figure 4 and Figure 5). The level of necrosis was low (7%) and consistent across cell lines. Under these treatment conditions, differential effects were observed on rates of cell proliferation and apoptosis, and these effects were mirrored in differences among proteins regulating the G1/S transition in the cell cycle and proteins involved in the intrinsic pathway of apoptosis induction. The finding that distinct response patterns among molecular subtypes of breast cancer cells were induced by 4-oxo-DHA was consistent with the expectation that multiple signaling pathways were accounting for the observed effects. To evaluate this premise, the protein expression data were uploaded into IPA as an unbiased data-driven expansion of the investigation.

As discussed in preceding paragraphs, given the rationale underlying the proteins that were chosen for analysis based on the work reported in [10], the fact that Figure 6 and Figure 7 highlight the mTOR signaling network of which AMPK and PI3K/AKT are components, was expected, and the identification of insulin receptor signaling is also a recognized regulator of mTOR network activity. However, the implication of autophagy, sirtuin signaling, endocannabinoid cancer inhibition, and HER-2 signaling in breast cancer were not anticipated and provided insights. Briefly, the activation status of these canonical pathways suggests: (1) that apoptosis induction by the intrinsic pathway, a primary driver of the anticancer activity (Table 3), may involve the induction of autophagy which can drive a cell to undergo apoptosis [25,26], (2) that expected effects of 4-oxo-DHA on PPAR activity may involve mediation via sirtuins [27], (3) that the metabolism of endogenous cannabinoids, which are metabolites of arachidonic acid, may be altered by 4-oxo-DHA in a manner that induces cancer inhibitory activity of the endocannabinoid signaling pathway, and (4) that the pattern of protein expression induced by 4-oxo-DHA overlaps with inhibition of HER-2 signaling in breast cancer. Moreover, the heatmap (Supplemental Figure S2) reflecting the comparative differences in the activation status of these canonical pathways across cell lines supports the concept that different molecular subtypes of breast cancer respond differently to the same metabolite of DHA. This idea is further supported by the heatmap of diseases and cell function (Figure 8). In addition, that heat map suggests that in vivo studies of the therapeutic use of DHA metabolites as adjuvants during and after cancer treatment should investigate effects mediated via modulation of the immune system, specifically T cell function and cell death regulation.

Given that canonical signaling analysis in IPA identified the inhibition of HER-2 signaling in breast cancer, the Analysis Match function in IPA was used to identify databases in the public domain that have been annotated in the IPA knowledge database for patterns of expression consistent with the effects of 4-oxo-DHA on MDAMB468, a poor prognosis molecular subtype of breast cancer with metastatic potential. That analysis was filtered for datasets in LINC in which targeted breast cancer treatment agents were evaluated. The molecular targets and therapeutic agents investigated are summarized in Table 5. Given that those agents induced patterns of gene expression consistent with the patterns of protein expression induced by 4-oxo-DHA, we suggest that this provides a platform by which to overlay nutrient and therapeutic agent mediated effects that will direct the selection of nutrient-therapeutic combinations to investigate as candidate PON treatment regimes.

**Figure 10:**
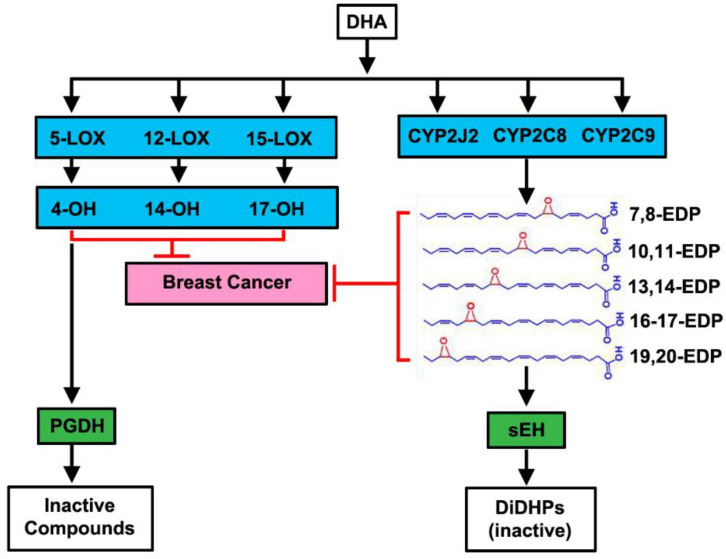
Schematic of the metabolism of DHA.

Herein, the focus has been on one metabolite of DHA in a series of proof-in-concept data-driven experiments to provide a foundation for PON. The use of this in vitro approach was purposeful in that the number of DHA metabolites that should be investigated is significant (Figure 10) and could be further expanded to over 100 metabolites if other N3FA and N6FA were considered. Thus, a facile screening platform will be required to make progress and this approach could be readily juxtaposed with human cancer cell line panels commonly used in drug development.

## 5. Conclusions

In summary, the evidence reported is consistent with anticancer effects of 4-oxo-DHA mediated primarily via induction of apoptosis via the intrinsic pathway. Breast cancer molecular subtypes vary in responsiveness to 4-oxo-DHA, and canonical pathways not previously associated with N3FA activity appear to participate in the inhibition of cancer cell accumulation in culture. The in vitro approach coupled with bioinformatics as reported herein can contribute to the identification of key N3FA species and the molecular targets by which they can be coupled with therapeutic strategies that collectively enhance breast cancer treatment efficacy and improve the prognosis for long term survival. This is the goal of precision onco-nutrition.

## Figures and Tables

**Table 1:** Effect of 4-oxo-DHA on Cell Proliferation and Apoptotic Cell Death.

Cell Lines	BT474		SKBR-3		MDAMB468	
ER	+		-		-	
PR	+		-		-	
HER2	+		+		-	
4-oxo-DHA (µM)	0	25	0	25	0	25
Cell proliferation						
Index (OD)	0.24 ± 0.01	0.23 ± 0.01 *	0.56 ± 0.01	0.50 ± 0.01 *	0.45 ± 0.01	0.37 ± 0.01 *
Rb^Ser780^ ratio	1.16 ± 0.08	0.45 ± 0.01*	0.83 ± 0.03	0.70 ± 0.02 *	3.21 ± 0.10	2.22 ± 0.07 *
Cyclin D1	2703 ± 66	2201 ± 42 *	2666 ± 185	2643 ± 142	2494 ± 50	1895 ± 60 *
P21	24.0 ± 0.9	34.4 ± 2.9 *	30.3 ± 3.6	45.4 ± 1.3 *	49.9 ± 1.6	63.4 ± 2.9 *
P27	45.5 ± 2.2	84.3 ± 3.8 *	17.1 ± 1.5	29.7 ± 1.8 *	44.0 ± 2.5	52.9 ± 1.4 *
Apoptosis						
Index (%)	3.0 ± 0.1	25.1 ± 0.5 *	3.1 ± 0.2	18.8 ± 0.5 *	3.0 ± 0.1	14.6 ± 0.3 *
Apaf-1	190 ± 6	183 ± 4	255 ± 9	304 ± 15 *	529 ± 7	521 ± 10
Bax	49.9 ± 1.8	32.5 ± 0.6 *	92.8 ± 5.1	130 ± 7 *	220 ± 15	248 ± 15
Bcl-2	231 ± 12	99.5 ± 2.7 *	47.8 ± 2.2	38.3 ± 3.7 *	538 ± 14	367 ± 23 *
Bax/Bcl-2	0.22 ± 0.01	0.33 ± 0.01 *	1.95 ± 0.06	3.62 ± 0.44 *	0.41 ± 0.04	0.68 ± 0.02 *
PARP89	63.3 ± 4.6	28.9 ± 1.1 *	51.6 ± 2.9	44.1 ± 6.5	84.0 ± 1.5	88.1 ± 2.4
PARP116	1225 ± 16	407 ± 5 *	1614 ± 40	1301 ± 132	1215 ± 25	973 ± 39 *
PARP89/116	0.05 ± 0.01	0.07 ± 0.01 *	0.03 ± 0.01	0.03 ± 0.01	0.07 ± 0.01	0.09 ± 0.01*

Values are means ± SEM (*n* = 8); Data were analyzed by Kruskal–Wallis rank test (* *p* < 0.05, 0 µM versus 25 µm 4-oxo-DHA).

**Table 2:** Effect of 4-oxo-DHA on Cell Transcription Factors.

Cell Lines	BT474		SKBR3		MDAMB468	
ER	+		-		-	
PR	+		-		-	
HER2	+		+		-	
4-oxo-DHA (µM)	0	25	0	25	0	25
PPARβ	153 ± 3	86 ± 2 *	130 ± 5	110 ± 2 *	198 ± 10	215 ± 8
PPARγ	45 ± 1	57 ± 1 *	94 ± 2	124 ± 3 *	126 ± 1	153 ± 2 *
GPR120	13.1 ± 0.3	22.6 ± 0.8 *	25.3 ± 1.1	25.5 ± 1.5	31.6 ± 1.1	41.8 ± 3.6 *
Hif-1α	179 ± 8	96 ± 4 *	398 ± 20	434 ± 48	468 ± 30	319 ± 35 *
SIRT-1	663 ± 18	304 ± 5 *	1228 ± 85	1266 ± 72	1021 ± 41	927 ± 34
GADD153	87 ± 3	43 ± 2 *	209 ± 23	274 ± 407	432 ± 28	338 ± 10 *
Ratios						
NF-κB p65^Ser536^	0.65 ± 0.04	0.45 ± 0.03 *	0.45 ± 0.02	0.50 ± 0.02	2.38 ± 0.12	1.28 ± 0.08 *
FOXO3a^Thr32^	3.32 ± 0.04	0.94 ± 0.19 *	1.92 ± 0.18	0.80 ± 0.11 *	5.97 ± 0.06	5.25 ± 0.09 *

Values are means ± SEM (*n* = 8); Data were analyzed by Kruskal–Wallis rank test (* *p* < 0.05, 0 µM versus 25 µm 4-oxo-DHA).

**Table 3:** Effect of 4-oxo-DHA on Growth Factor Signaling.

Cell Lines	BT474		SKBR3		MDAMB468	
ER	+		-		-	
PR	+		-		-	
HER2	+		+		-	
4-oxo-DHA (µM)	0	25	0	25	0	25
IGF-1R	80 ± 5	38 ± 2 *	61 ± 4	50 ± 1 *	296 ± 3	236 ± 2 *
PI3Kp110	174 ± 5	129 ± 3 *	294 ± 16	228 ± 16 *	192 ± 1	158 ± 3 *
Ratios						
IRS1^Ser636/639^	0.52 ± 0.02	0.27 ± 0.01 *	0.77 ± 0.05	0.79 ± 0.06	0.60 ± 0.02	0.59 ± 0.02
AMPK^Thr172^	0.06 ± 0.01	0.25 ± 0.01 *	0.07 ± 0.01	0.12 ± 0.01 *	0.08 ± 0.01	0.11 ± 0.01 *
Akt^Ser473^	3.29 ± 0.13	0.78 ± 0.03 *	3.23 ± 0.23	1.01 ± 0.08 *	7.68 ± 0.29	6.50 ± 0.26 *
mTOR^Ser2448^	0.61 ± 0.02	0.23 ± 0.01 *	0.17 ± 0.01	0.16 ± 0.01	0.25 ± 0.02	0.20 ± 0.01 *
Raptor^Ser792^	0.08 ± 0.01	0.15 ± 0.01 *	0.05 ± 0.01	0.06 ± 0.01	0.08 ± 0.01	0.10 ± 0.01 *
PRAS40^Thr246^	2.79 ± 0.08	1.80 ± 0.07 *	1.43 ± 0.06	1.26 ± 0.03	2.47 ± 0.06	2.05 ± 0.01 *
P70S6K^Thr389^	0.26 ± 0.01	0.10 ± 0.01 *	0.55 ± 0.17	0.10 ± 0.01 *	0.93 ± 0.03	0.67 ± 0.03 *
4E-BP1^Thr37/46^	1.30 ± 0.09	0.88 ± 0.03 *	1.62 ± 0.27	1.39 ± 0.16	0.89 ± 0.04	0.65 ± 0.02 *

Values are means ± SEM (*n* = 8); Data were analyzed by Kruskal–Wallis rank test (* *p* < 0.05, 0 µM versus 25 µm 4-oxo-DHA).

**Table 4:** Effect of 4-oxo-DHA on Lipid Metabolism.

Cell Lines	BT474		SKBR-3		MDAMB-468	
ER	+		-		-	
PR	+		-		-	
HER2	+		+		-	
4-oxo-DHA (µM)	0	25	0	25	0	25
FASN	787 ± 22	552 ± 3 *	2081 ± 86	2150 ± 34	1141 ± 82	1132 ± 74
HMGCR	469 ± 8	396 ± 9 *	275 ± 22	309 ± 33	566 ± 20	432 ± 42 *
SREBP-1	297 ± 11	123 ± 3 *	568 ± 25	399 ± 8 *	575 ± 17	520 ± 28
ACC^Ser79^ ratio	0.36 ± 0.03	0.75 ± 0.08 *	0.28 ± 0.03	1.13 ± 0.35 *	0.83 ± 0.02	1.07 ± 0.04 *

Values are mean ± SEM (*n* = 8); Data were analyzed by Kruskal–Wallis rank test (* *p* < 0.05, 0 µM versus 25 µm 4-oxo-DHA).

**Table 5:** IPA Analysis match for the 4-oxo DHA-induced expression profile in MDAMB468.

Target Gene ^1^	Treatment ^2^	Z-Score ^3^	Accession ID
mTOR	AZD8055	23.88	GSE70138
multiple targets	Celastrol	16.15	GSE70138
CDK1; 2	CGP60474	15.67	GSE70138
CDK	AT7519	14.17	GSE70138
CDK	AZD5438	13.36	GSE70138
CDK9	Alvocidib	14.17	GSE70138
JNKs	CC401	15.67	GSE70138
MEK	AZD8330	14.94	GSE70138
IKKbeta; alpha ^2^	BMS345541	14.17	GSE70138

^1^ mTOR, mammalian target of rapamycin; CDK1, cyclin dependent kinase 1; CDK2, cyclin dependent kinase-2; JNK, jun N terminal kinase; MEK, mitogen activated protein kinase kinase; IKKbeta or IKKalpha, inhibitor of nuclear factor kappa-B kinase subunit. ^2^ Treatments: by chemical names andPubChem CID: AZD8055, [5-[2,4-bis[(3S)-3-methylmorpholin-4-yl]pyrido [2,3-d]pyrimidin-7-yl]-2-methoxyphenyl]methanol, Compound CID: 25262965; Celastrol; Tripterin(e) (2R,4aS,6aR,6aS,14aS,14bR)-10-hydroxy-2,4a,6a,6a,9,14a-hexamethyl-11-oxo-1,3,4,5,6,13,14,14b-octahydropicene-2-carboxylic acid, Compound CID: 122724; CGP60474,-[[4-[2-(3-chloroanilino)pyrimidin-4-yl]pyridin-2-yl] amino]propan-1-ol, Compound CID: 644215; AT7519,4-[(2,6-dichlorobenzoyl)amino]-N-piperidin-4-yl-1H-pyrazole-5-carboxamide, Compound CID: 11338033; AZD5438,4-(2-methyl-3-propan-2-ylimidazol-4-yl)-N-(4-methylsulfonylphenyl)pyrimidin-2-amine, Compound CID: 16747683; Alvocidib; Flavopiridol, 2-(2-chlorophenyl)-5,7-dihydroxy-8-[(3S,4R)-3-hydroxy-1-methylpiperidin-4-yl]chromen-4-one, Compound CID: 5287969; CC4013-[3-(2-piperidin-1-ylethoxy)phenyl]-5-(1H-1,2,4-triazol-5-yl)-1H-indazole, Compound CID: 10430360; AZD8330,2-(2-fluoro-4-iodoanilino)-N-(2-hydroxyethoxy)-1,5-dimethyl-6-oxopyridine-3-carboxamide, Compound CID: 16666708; BMS345541, N′-(1,8-dimethylimidazo[1,2-a]quinoxalin-4-yl)ethane-1,2-diamine, Compound CID: 9813758. ^3^ Z-score. IPA computed a z-score for the match of the “query” signature against the signatures of all other analyses. The larger the z-score the stronger the match.

## Data Availability

The data presented in this study are available on request from the corresponding author.

## Supplementary Materials

The following are available online at https://www.mdpi.com/article/10.3390/cancers14010157/s1, Figure S1: Electropherogram profiles of key primary antibodies used for western blotting; Figure S2: Dose- and time-dependent effects of 4-oxo-DHA on the growth of five subtype human breast cancer cells; Figure S3: Heatmaps of canonical pathways associated with 4-oxo-DHA treatment across common molecular subtypes of breast cancer title; Table S1: Primary antibodies; Table S2: Disease and Function Annotations from IPA; Table S3: Analysis match results.
