# Supplementary material for: Building a Foundation for Precision Onco-Nutrition: Docosahexaenoic Acid and Breast Cancer
Source: Cancers (Basel). 2021 Dec 29;14(1):157. doi: 10.3390/cancers14010157 (PMC8750666; doi:10.3390/cancers14010157)
Supplement: Supplementary file 1 [file cancers-14-00157-s001.zip › supplement.pdf]

## Supplemental Data

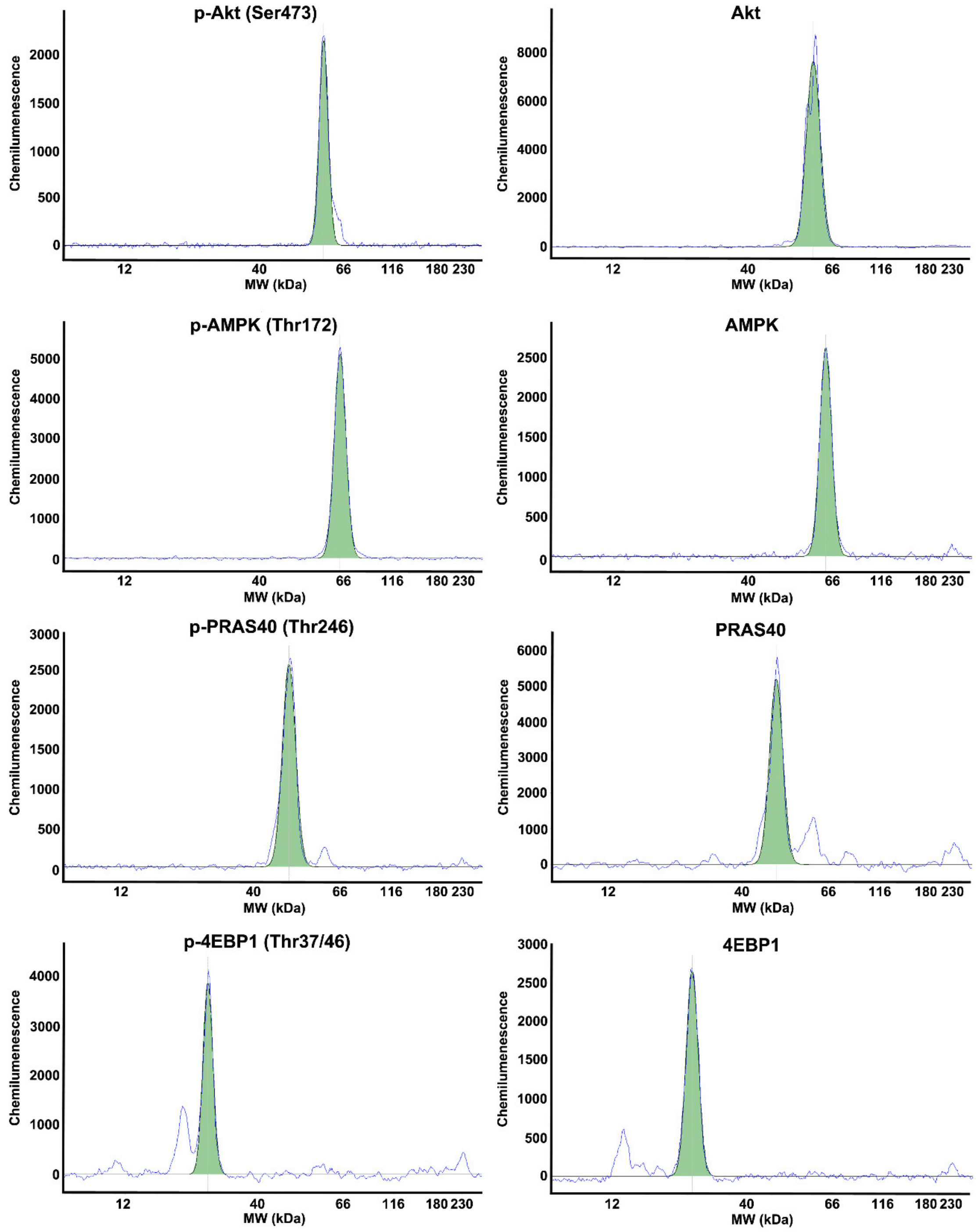

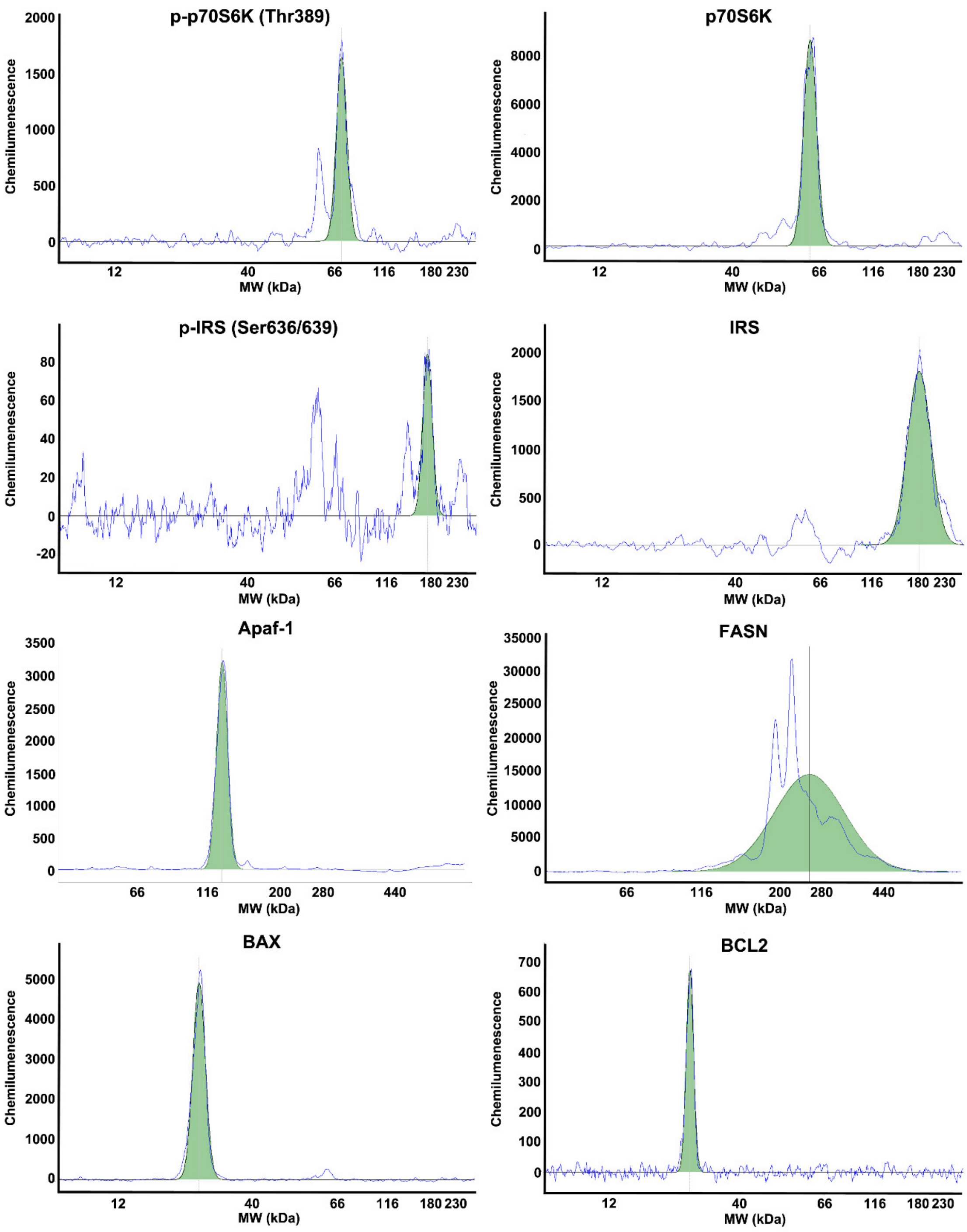
p-p70S6K (Thr389)
p70S6K
p-IRS (Ser636/639)
IRS
Apaf-1
FASN
BAX
BCL2
Chemilumenescence
MW (kDa)
12
40
66
116
180 230
200
280
440

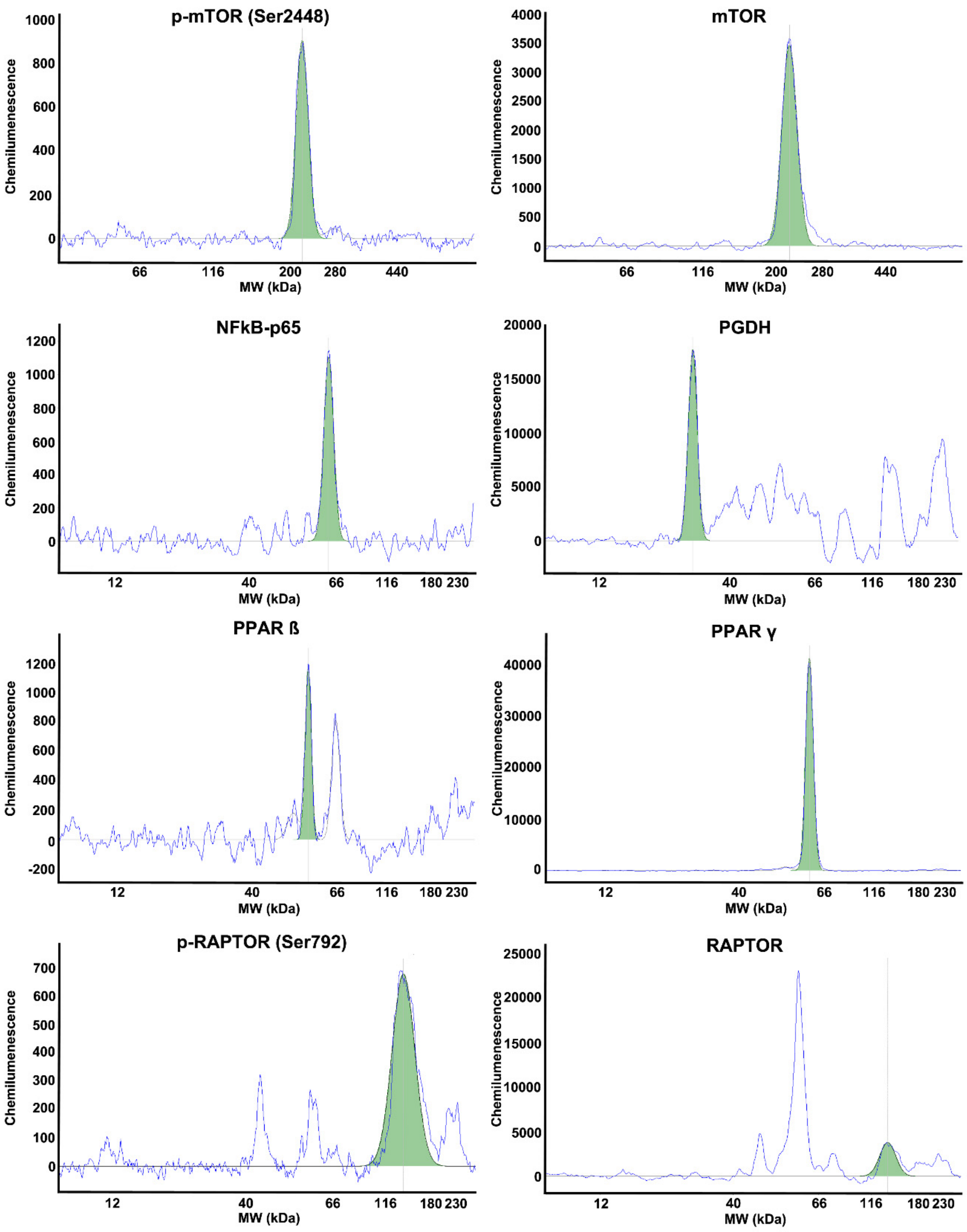
p-mTOR (Ser2448)
mTOR
NFkB-p65
PGDH
PPAR ß
PPAR γ
p-RAPTOR (Ser792)
RAPTOR
Chemilumenescence
MW (kDa)
66
116
200
280
440
12
40
180 230
1000
800
600
400
200
0
4000
3500
3000
2500
2000
1500
500
1200
20000
15000
10000
5000
-200
40000
30000
700
300
100
25000

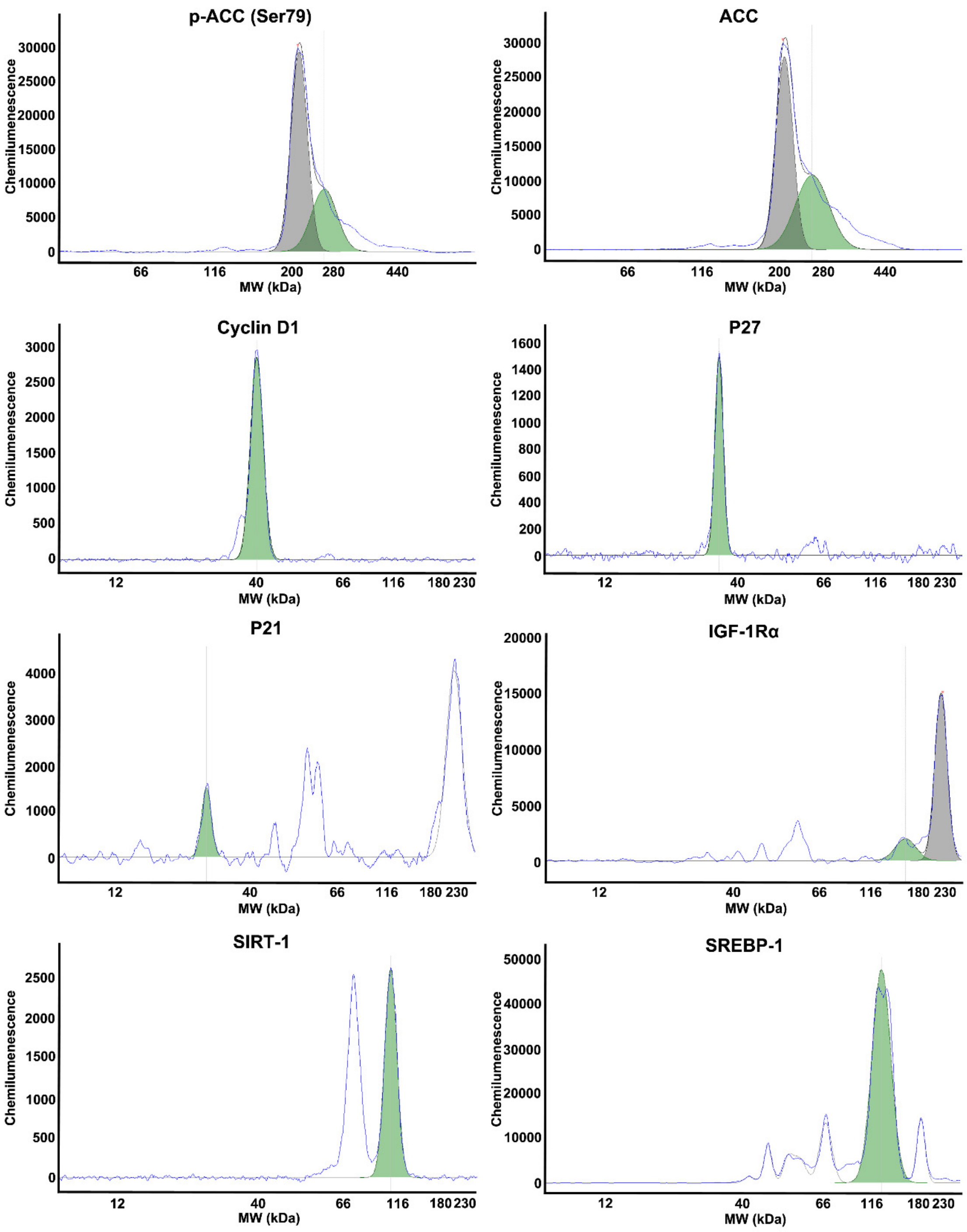


**Figure S1.** Electropherogram profiles of key primary antibodies used for western blotting.

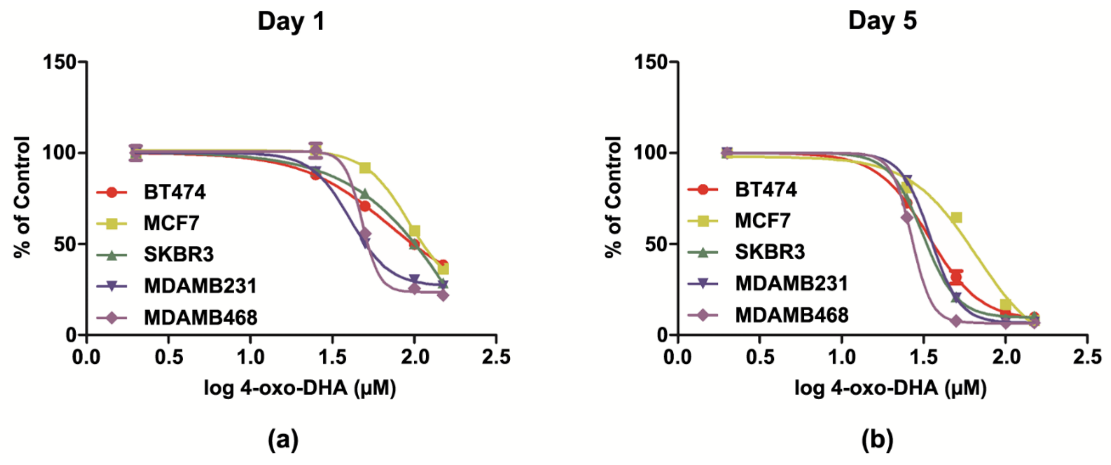


**Figure S2**. Dose- and time-dependent effects of 4-oxo-DHA on the growth of five subtype human breast cancer cells (BT474, MCF7, SKBR3, MDAMB231, and MDAMB468) graphed using the half maximal inhibitory concentration (IC50) as percent of untreated control for 1 and 5 days treatment.

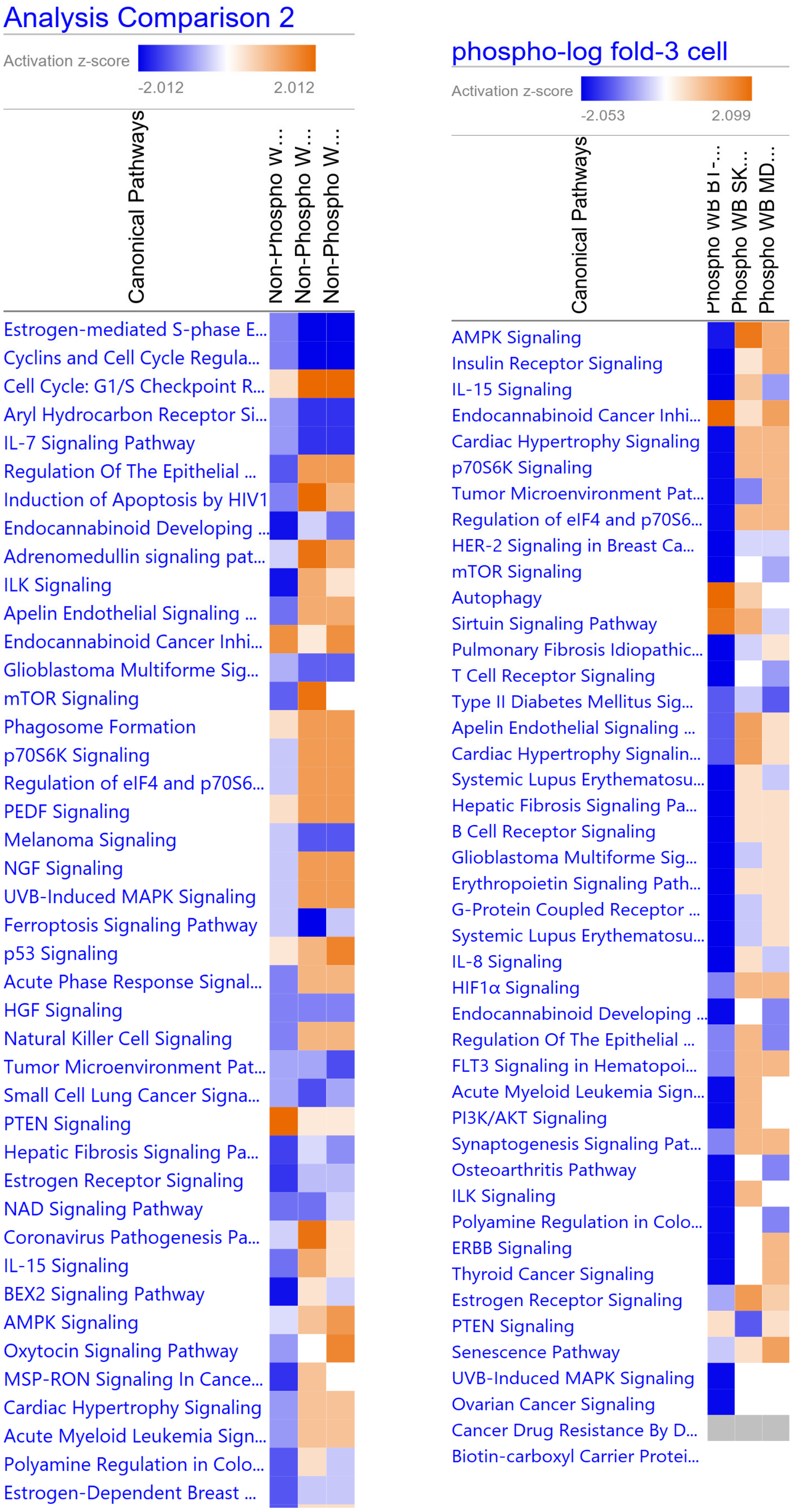


**Figure S3.** Heatmaps of canonical pathways associated with 4-oxo-DHA treatment across common molecular subtypes of breast cancer.

**Table S1.** Primary antibodies.

| Antibody | Vendor | Catalog # |
|---|---|---|
| phospho-ACC$^{Ser79}$ | Cell Signaling | 3661 |
| ACC | Cell Signaling | 3676 |
| β Actin | Sigma Aldrich | A5441 |
| phospho-Akt$^{Ser473}$ | Cell Signaling | 4058 |
| Akt | Cell Signaling | 9272 |
| phospho-AMPK$^{Thr172}$ | Cell Signaling | 2535 |
| AMPK | Cell Signaling | 2603 |
| Apaf-1 | Cell Signaling | 8723 |
| Bax | BD Biosciences | 556467 |
| Bcl-2 | BD Biosciences | 610538 |
| cyclin-D1 | BD Biosciences | 556470 |
| phospho-4E-BP1$^{Thr37/46}$ | Cell Signaling | 9459 |
| 4E-BP1 | Cell Signaling | 9452 |
| FASN | Cell Signaling | 3180 |
| phospho-FOXO1$^{Thr24}$ & FOX03$^{Thr32}$ | Cell Signaling | 9464 |
| FOXO1 & FOX03 | Cell Signaling | 9462 |
| GADD153 | Novus Biologicals | NB600-1335 |
| GPR120 | Novus Biologicals | NBP1-00858 |
| Hif-1$\alpha$ | Novus Biologicals | NB100-105 |
| HMGCR | Santa Cruz | sc-27578 |
| IGF1R$\alpha$ | Santa Cruz | sc-712 |
| IGFR-1 | Santa Cruz | sc-7952 |
| phospho-IRS$^{Ser636/639}$ | Cell Signaling | 2388 |
| IRS | Cell Signaling | 2382 |
| phospho-mTOR$^{Ser2448}$ | Cell Signaling | 2971 |
| mTOR | Cell Signaling | 2972 |
| NFkB-p65 | Cell Signaling | 3987 |
| p21Cip1 | Santa Cruz | sc-6246 |
| p27Kip1 | BD Biosciences | 610241 |
| Phospho-p70S6K$^{Thr389}$ | Cell Signaling | 9205 |
| p70S6K | Cell Signaling | 2708 |
| PARP | Cell Signaling | 9542 |
| PGDH | Santa Cruz | sc-48908 |
| PI3Kp110 | Cell Signaling | 4255 |
| PPARβ, γ | Santa Cruz | sc-365615 |
| phospho-PRAS40$^{Thr246}$ | Cell Signaling | 2997 |
| PRAS40 | Cell Signaling | 2691 |
| phospho-Raptor$^{Ser792}$ | Cell Signaling | 2083 |
| Raptor | Cell Signaling | 2280 |
| phospho-RB$^{Ser780}$ | Cell Signaling | 9307 |
| RB | Cell Signaling | 9313 |
| SIRT-1 | Cell Signaling | 9475 |
| SREBP-1 | Santa Cruz | sc-365513 |

**Table S2.** Disease and Function Annotations from IPA.

| Type | Cell Line | Diseases or Functions Annotation | z-Score | Predicted | *p*-value | # Molecules |
|---|---|---|---|---|---|---|
| NP | BT474 | Cell proliferation of breast cancer cell lines | -3.524 | Decreased | 2.88E-25 | 22 |
| | BT474 | Proliferation of lymphocytes | -3.157 | Decreased | 4.49E-16 | 18 |
| | BT474 | Proliferation of B lymphocytes | -3.087 | Decreased | 2.8E-15 | 13 |
| | BT474 | Proliferation of immune cells | -2.958 | Decreased | 6.38E-17 | 19 |
| | BT474 | Growth of tumor | -2.903 | Decreased | 7.09E-27 | 27 |
| | SKBR3 | Inflammation of body cavity | -2.681 | Decreased | 8.56E-12 | 17 |
| | SKBR3 | Proliferation of hepatocytes | -2.478 | Decreased | 4.27E-12 | 9 |
| | SKBR3 | Cell cycle progression of tumor cell lines | -2.396 | Decreased | 2.22E-22 | 17 |
| | SKBR3 | Cell proliferation of fibroblasts | -2.293 | Decreased | 1.17E-12 | 12 |
| | SKBR3 | Inflammation of absolute anatomical region | -2.263 | Decreased | 7.84E-13 | 19 |
| | MDAMB 468 | Colony formation of cells | -2.749 | Decreased | 5.69E-25 | 22 |
| | MDAMB 468 | Migration of cells | -2.702 | Decreased | 5.76E-21 | 29 |
| | MDAMB 468 | Senescence of tumor cell lines | 2.559 | Increased | 6.99E-12 | 8 |
| | MDAMB 468 | Differentiation of adipocytes | 2.513 | Increased | 4.86E-15 | 13 |
| | MDAMB 468 | Proliferation of hepatocytes | -2.478 | Decreased | 4.27E-12 | 9 |
| P | BT474 | Activation of blood cells | -2.449 | Decreased | 1.26E-06 | 7 |
| | BT474 | Consumption of oxygen | 2.425 | Increased | 3.69E-09 | 6 |
| | BT474 | Apoptosis of tumor cell lines | 2.396 | Increased | 2.74E-14 | 13 |
| | BT474 | Size of animal | -2.364 | Decreased | 1.2E-09 | 6 |
| | BT474 | Secretion of molecule | -2.335 | Decreased | 9.18E-08 | 7 |
| | SKBR3 | Proliferation of B lymphocytes | 2.213 | Increased | 1.54E-06 | 5 |
| | SKBR3 | Organismal death | -2.14 | Decreased | 2.9E-06 | 10 |
| | SKBR3 | Concentration of lipid | -2.044 | Decreased | 6.35E-09 | 9 |
| | SKBR3 | Apoptosis of prostate cancer cell lines | 2.011 | Increased | 1.4E-11 | 7 |
| | MDAMB 468 | Cell death of immune cells | -2.804 | Decreased | 5.36E-14 | 11 |
| | MDAMB 468 | Cell death of central nervous system cells | -2.406 | Decreased | 1.6E-11 | 8 |
| | MDAMB 468 | Apoptosis of leukocyte cell lines | -2.403 | Decreased | 5.25E-12 | 7 |
| | MDAMB 468 | Quantity of monosaccharide | -2.224 | Decreased | 5.86E-09 | 7 |
| | MDAMB 468 | Invasion of breast cancer cell lines | -2.219 | Decreased | 1.31E-07 | 6 |
| R | BT474 | Immune response of cells | 2.506 | Increased | 9.75E-10 | 9 |
| | BT474 | Activation of lymphocytes | -2.442 | Decreased | 3.78E-09 | 8 |
| | BT474 | Size of animal | -2.364 | Decreased | 3.44E-09 | 6 |
| | BT474 | Secretion of molecule | -2.335 | Decreased | 3.24E-07 | 7 |
| | BT474 | Concentration of lipid | -2.287 | Decreased | 2.53E-11 | 11 |
| | SKBR3 | Immune response of cells | 2.506 | Increased | 9.75E-10 | 9 |
| | SKBR3 | Quantity of protein in blood | -2.433 | Decreased | 1.62E-07 | 7 |

| | | | | | |
|---|---|---|---|---|---|
| SKBR3 | Proliferation of neuroglia | -2.215 | Decreased | 1.03E-07 | 5 |
| SKBR3 | Accumulation of lipid | -2.213 | Decreased | 2.65E-07 | 6 |
| SKBR3 | Oxidation of fatty acid | 2.201 | Increased | 1.08E-10 | 7 |
| MDAMB 468 | Immune response of cells | 2.506 | Increased | 9.75E-10 | 9 |
| MDAMB 468 | Activation of lymphocytes | -2.442 | Decreased | 3.78E-09 | 8 |
| MDAMB 468 | Size of animal | -2.364 | Decreased | 3.44E-09 | 6 |
| MDAMB 468 | Secretion of molecule | -2.335 | Decreased | 3.24E-07 | 7 |
| MDAMB 468 | Concentration of lipid | -2.287 | Decreased | 2.53E-11 | 11 |

NP, non-phosphorylated; P, phosphorylated; R, ratio.

**Table S3:** Analysis Match results (Separate excel file).
